# Isolated spinal osteomyelitis caused by *Nocardia farcinica* in an immunocompetent patient: A case report and literature review

**DOI:** 10.1097/MD.0000000000042797

**Published:** 2025-09-05

**Authors:** Yihui Liu, Yuanpeng Yue, Ce Dong, Zhenyu Wang

**Affiliations:** a Department of Spinal Surgery, The First Hospital of Jilin University, ChangChun, Jilin Province, China; b Department of Spinal Surgery, Jilin University, Changchun, Jilin Province, China.

**Keywords:** case report, immunocompetent, *Nocardia farcinica*, osteomyelitis

## Abstract

**Rationale:**

*Nocardia* spp. are opportunistic pathogens that invade the human body via respiratory inhalation or direct skin wounds. Spinal nocardial osteomyelitis is a rare disease with only a few cases reported to date. To the best of our knowledge, this is the second case of spinal osteomyelitis caused by *Nocardia farcinica*.

**Patient concerns and diagnoses:**

A 12-year-old immunocompetent girl was diagnosed with spinal osteomyelitis. The causative agent has been confirmed as *N farcinica* by metagenomic next-generation sequencing analysis of vertebral biopsy tissue in December 2022. It was noteworthy that the onset of the disease in this patient was insidious and the symptoms were atypical, which differed from previously reported cases.

**Interventions:**

Trimethoprim/sulfamethoxazole was given first, showing good clinical effects. To clarify the changes in the patient’s condition, we performed magnetic resonance imaging (MRI) and computed tomography examinations on the patient in August 2023.

**Outcomes:**

After 2 months of medication, the patient’s clinical symptoms completely disappeared. The results of the latest computed tomography and MRI scans showed the formation of hardened bone in the area of the L2 vertebral body bone erosion, and MRI showed a significant reduction in the abnormal signal range of the L2 vertebral body, which was considered cured.

**Lessons:**

This study suggests that *N farcinica*, a rare pathogen, can present with atypical symptoms and can easily be misdiagnosed in immunocompromised patients. Its diagnosis relies on advanced testing techniques, and determining the nature of the pathogen is of great significance for a clear diagnosis. Moreover, early, sufficient, and comprehensive treatment with sulfonamide antibiotics or combination therapy usually results in a good prognosis.

## 1. Introduction

*Nocardia* spp is a genus of aerobic, branching, gram-positive, partially acid-fast, rod-shaped bacteria that are widely distributed in natural soil, decaying organisms, seawater, fresh water, and dust.^[1]^ Nocardia spp can be inhaled through the respiratory tract or directly invade the human body through skin wounds, causing localized or disseminated purulent or granulomatous lesions. Thus, the most common sites of infection are the lungs (40%), brain (20–40%), and the skin, and bone involvement is relatively less common.^[2,3]^ About 80% of nocardiosis presentations are abscesses of the brain, pulmonary abscesses or disseminated infection, while the skin is affected in the remaining 20% of patients.^[4]^ To the best of our knowledge, this is the second reported case of primary spinal osteomyelitis caused by *Nocardia farcinica.* We present a case of a patient who was successfully treated with oral trimethoprim/sulfamethoxazole (TMP-SMZ). In addition, we reviewed existing literature on spinal nocardial osteomyelitis. Informed consent was obtained from all the patients for this study.

## 2. Case report

A 12-year-old girl sought medical attention at a regional hospital in November 2022 because of worsening low back pain and right lower limb pain for 1 month. Her medical history was uneventful and she had no history of other infectious diseases or tumors.

**Figure 1.**
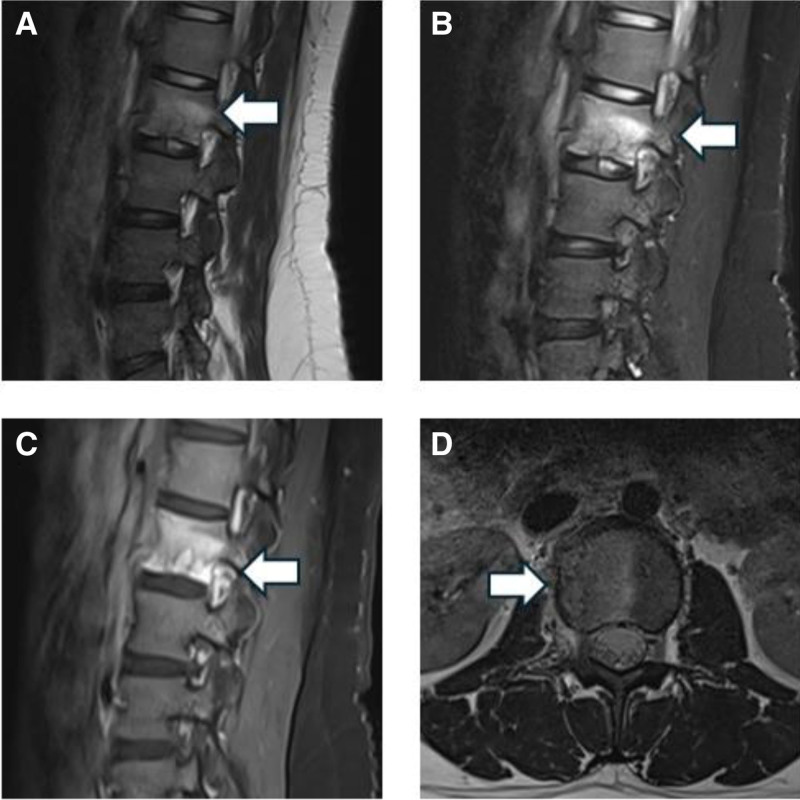
MRI images obtained before admission. Sagittal Gd-enhanced T1-weighted (A), T2-weighted (B), T2-weighted fat-saturation (C), and axial Gd-enhanced T2-weighted images (D) showed patchy abnormal signal shadows on the L2 vertebral body. MRI = magnetic resonance imaging.

**Figure 2.**
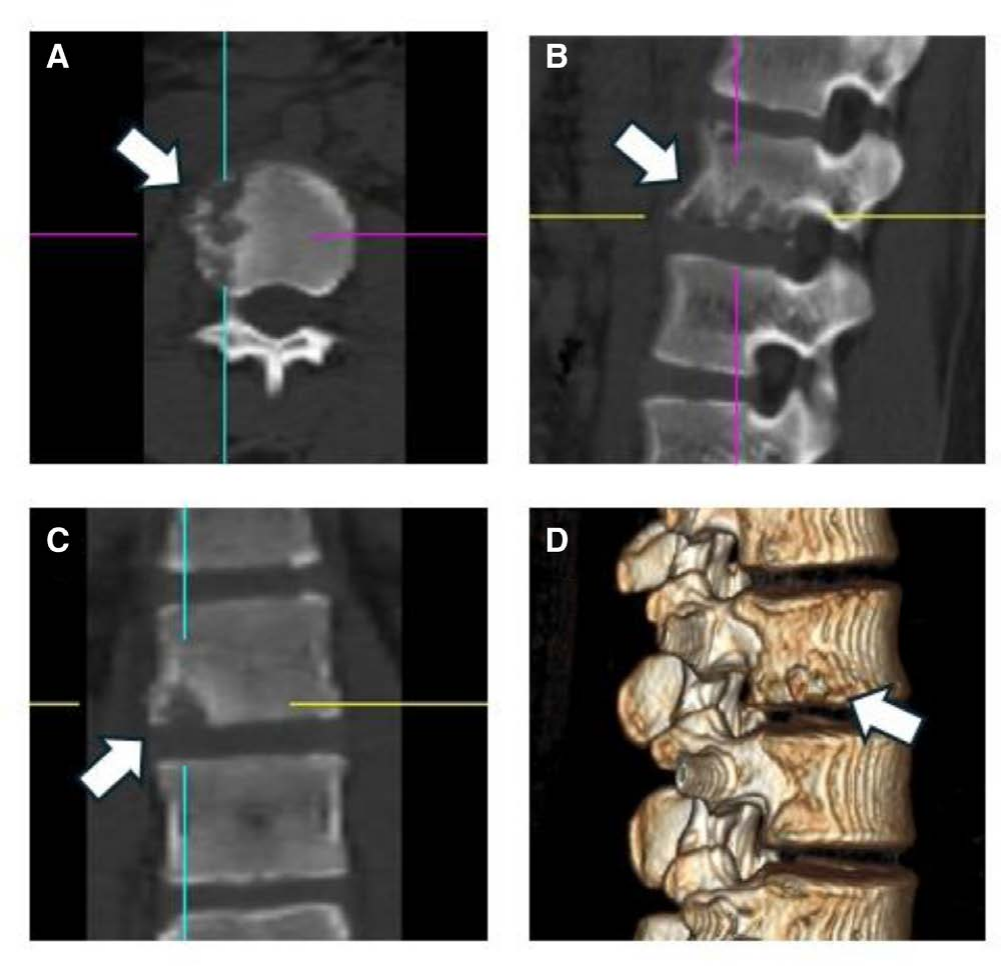
CT images obtained before admission. Horizontal (A), sagittal (B), and coronal (C) CT images and 3D reconstruction (D) show the osteolytic changes in the lesion bone. CT = computed tomography.

On admission, her body temperature was normal, but she had severe back pain. With intermittent pain, the patient was unable to stand when the pain was severe. The patient’s symptoms were relieved when lying flat on the bed, but worsened significantly after changing positions. No significant correlation was found between leg pain and low back pain. Furthermore, neurological examination showed that, except for a slight decrease in shallow sensation in the front of the right thigh, there were no other changes in neurological function, her vital signs were within normal limits. Head, heart, lung and abdominal were also normal. The patient’s erythrocyte sedimentation rate was slightly faster at 36 mm/h (reference range: 0–20 mm/h), with a high-sensitivity C-reactive protein level of 5.55 mg/L (reference range: 0.0–1.0 mg/L). Table 1 presents the relevant laboratory test values and their reference range for this specific patient. No abnormalities were observed in white blood cell count, neutrophil percentage, tuberculosis, brucellosis test, procalcitonin level, tumor marker test, or other test results. The urine and blood culture results were negative. Lumbar computed tomography indicated local bone destruction on the right side of the L2 vertebral body (Fig. 2), whereas lumbar magnetic resonance imaging (MRI) showed patchy abnormal signal shadows on the L2 vertebral body (Fig. 1).

**Table 1:** Specific laboratory test values and normal range of this patient upon admission.

Items	Specific values (reference range)	Unit
WBC	0.64 (3.50–9.50)	10^9^/L
NE%	0.64 (0.40–0.75)	–
LY%	0.29 (0.20–0.50)	–
MO%	0.07 (0.03–0.10)	–
NE#	5.72 (1.80–6.30)	10^9^/L
LY#	2.60 (1.10–3.20)	10^9^/L
MO#	0.59 (0.10–0.60)	10^9^/L
EO#	0.08 (0.02–0.52)	10^9^/L
BA#	0.02 (0.00–0.06)	10^9^/L
PCT	<0.05 (0–0.05)	ng/mL
ESR	36 (0–20)	mm/1 h
CRP	5.55 (0–1.0)	mg/L

BA# = absolute basophil count, CRP = C-reactive protein, EO# = absolute eosinophil count, ESR = erythrocyte sedimentation rate, LY% = lymphocyte percentage, LY# = absolute lymphocyte count, MO% = monocyte percentage, MO# = absolute monocyte count, NE% = neutrophil percentage, NE# = absolute neutrophil count, PCT = procalcitonin, WBC = white blood cell.

Given that the nature of the lesion could not be determined based on the abovementioned tests, we performed percutaneous vertebral biopsy of the patient. Subsequently, metagenomic next-generation sequencing was performed, which indicated the presence of *N farcinica*. We then empirically administered TMP-SMZ and guided the patient to apply TMP-SMZ for 6 months.

**Figure 3.**
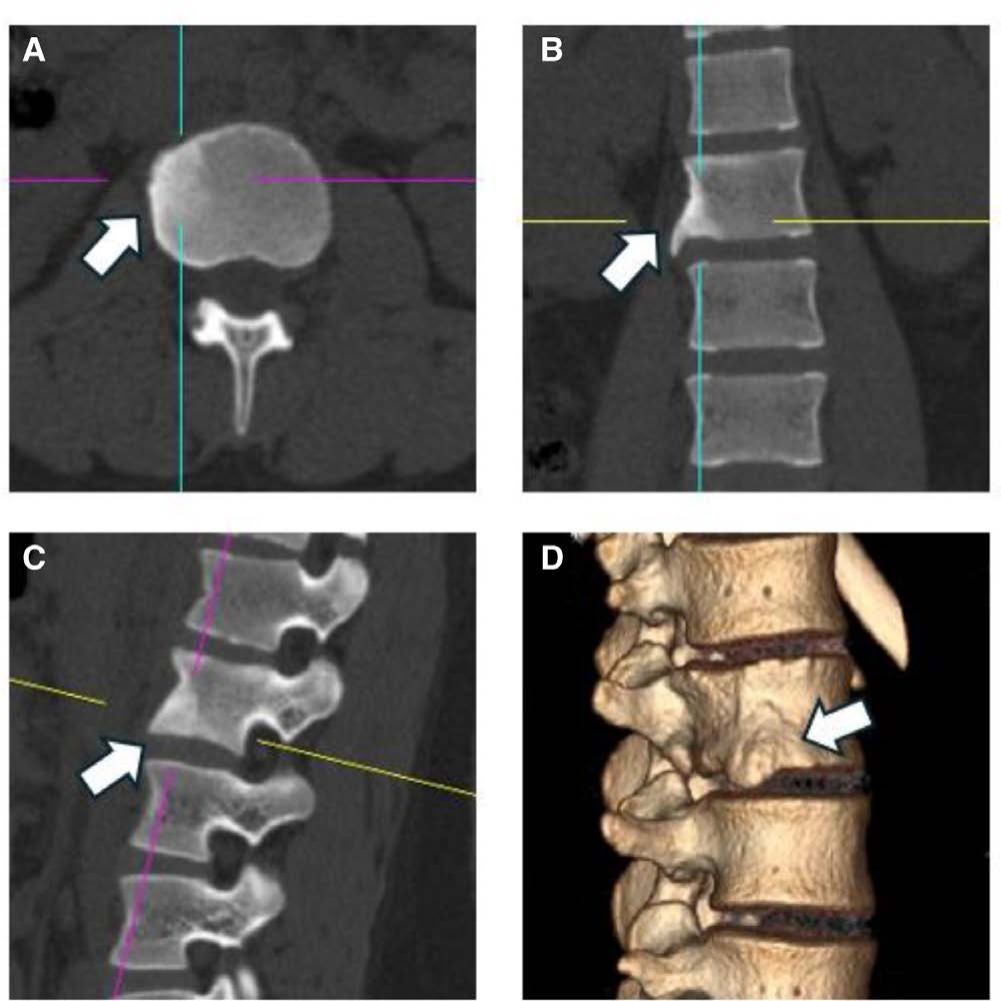
CT images obtained after discharge nearly 1.5 yr. Horizontal (A), sagittal (B), and coronal (C) CT images and 3D reconstruction (D) show that bone sclerosis has formed at the site of the lesion. CT = computed tomography.

**Figure 4.**
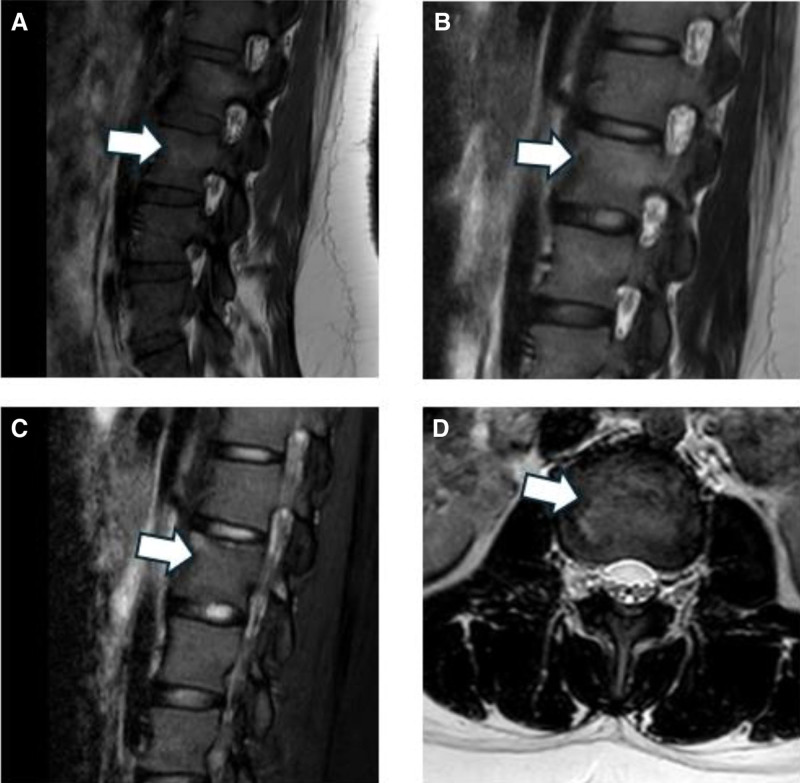
MRI images obtained after discharge nearly 1.5 yr. Sagittal T1-weighted (A), T2-weighted (B), T2-weighted fat-saturation (C), and axial T2-weighted images showed patchy abnormal signal shadows the significant reduction in the range of abnormal signals. MRI = magnetic resonance imaging.

After 50 days, her symptoms gradually disappeared, enabling independent ambulation. She underwent her first post-discharge follow-up. MRI showed a decrease in abnormal signals in the L2 vertebral body. During a 6-month telephone follow-up, her symptoms completely disappeared despite only 2 months of medication treatment. The final follow-up, conducted 1.5 years post-discharge (July 2024), showed no symptom recurrence. Computed tomography imaging (Fig. 3) indicated the formation of hardened bone in the area of the L2 vertebral body bone erosion, and MRI (Fig 4) showed a significant reduction in the abnormal signal range of the L2 vertebral body, which was considered cured.

## 3. Discussion

*Nocardia* spp are not a normal flora in the human body, and its infection is opportunistic.^[5]^ Immunodeficiency is the main risk factor for *Nocardia* spp, and the immune response is driven mainly by cell-mediated immunity through CD8+ T cells. Thus, individuals with acquired immunodeficiency syndrome, organ transplantation, malignant tumors, diabetes, alcoholism, or a long-term smoking history^[6]^ are at a higher risk of infection, and *Nocardial* infection is less common in individuals with normal immune function.^[7]^ The age of the patient in this case was 12 years, and there are currently no other known susceptibility factors, except for the possibility of an increased risk of infection due to incomplete development of the immune system at this age. However, some studies suggest that unidentified primary or genetic immune deficiencies may exist in patients with normal immunity, thereby increasing the risk of infection.^[6]^

Given the rarity of spinal osteomyelitis caused by Nocardial invasion of *Nocardia* spp, the number of published cases has been limited. We retrospectively analyzed 20 published English articles on nocardial spinal osteomyelitis. Most of these patients were male (75%, 15/20), with an average age of 47.4 ± 14.2 years. Most of these patients had risk factors such as immunodeficiency (18/20), including cancer, history of transplant, acquired immunodeficiency syndrome, and alcoholism. Clinical symptoms are often atypical and may include pain (70%, 14/20), neurological impairment (55%, 11/20), and fever (40%, 8/20). Furthermore, 60% (12/20) of patients had a clear history of primary infections, such as gardening experience, previous history of *Nocardial* infection (skin, lungs, bacteremia, etc), or a history of spinal surgery. Inflammatory indicators in patients often increase during severe infections, and most are accompanied by the formation of abscesses in different parts of the body. This finding suggests that the onset of Nocardial vertebral osteomyelitis is insidious and lacks diagnostic generality. Accordingly, it can be easily missed or misdiagnosed as lumbar disc herniation, lumbar spinal stenosis, or other infectious lesions, and the disease is detected relatively late. Therefore, improving our understanding of at risk individuals who are already susceptible to *Nocardia* spp with atypical symptoms is beneficial for faster diagnosis and treatment of *Nocardial* infection and, in turn, improving patient prognosis. Table 2 summarizes the basic information, main symptoms, treatment, and clinical outcomes of the historical cases. It is worth noting that the patient reported in this study is the youngest among all patients with nocardial spinal osteomyelitis, with no clear primary infection route and no risk factors for nocardial infection, such as immunodeficiency, atypical clinical symptoms, and neurological symptoms, which is extremely rare in previous reports. Pathogenic bacteria were finally identified in the patient through percutaneous vertebral puncture, and the patient then underwent drug treatment for only 2 months, with basic relief of pain symptoms. The last follow-up, 1.5 years post-discharge (July 2024), indicated complete resolution of symptoms and clinical recovery. However, the abnormal signal area of the lumbar spine persisted, indicating that imaging changes had a relatively lagging effect.

**Table 2:** Cases of spinal nocardial osteomyelitis reported in literature.

Time	Age/sex	Predisposing factors	Presentation	Infection segment	Type of infection	Surgical procedures	Antibiotics	Outcome
2023^[15]^	52/M	Greening workers	Headache, fever	L2–4	*Nocardia nova*	No	AMK → IPM/CA → IPM/CA + TMP/SMZ	Good
2023^[16]^	44/F	Pneumonia, Whole body dissemination	Back pain, cough, fever	L1–4	*Nocardia asteroides*	No	IPM/CA + TMP/SMZ → TMP/SMZ	Good
2022^[17]^	54/F	Lumbar surgery, spinal osteomyelitis caused by bacteria	Neck pain and right arm weakness	C4–5	*Nocardia nova*	Discectomy and washout	AMK → TMP/SMZ	Good
2022^[18]^	68/M	Localized application of glucocorticoids	Descending palsy, respiratory failure	C4	*Nocardia abscessus*	NA	NA	Good
2022^[19]^	37/M	HIV, pulmonary tuberculosis	Back pain, lumbar sacral nerve damage	T6–11	*Nocardia beijingensis*	No	TMP/SMZ + IPM → TMP/SMZ + IPM + CRO	Good
2021^[20]^	44/ML4–5 osteodiscitis	Nocardia bacteremia, L4–5 osteodiscitis	Low back pain	L4–5	*Nocardia nova*	No	TMP-SMZ + IPM/CA → TMP-SMZ	Good
2013^[21]^	69/M, long time, prednisone	Skin abscesses, pulmonary tuberculosis	Back pain	T1–9	*Nocardia brasiliensis*	Laminectomy and washout	MH + TMP-SMZ	Good
2007^[14]^	27/F	Renal transplant, hemodialysis, use of immunodepressants, pulmonary nocardiosis	Abdominal and low back pain, fever	L4–5	*Nocardia nova*	Incision and drainage of abscess	AMK + IMP + TMP/SMZ → AMX + IMP + TMP/SMZ → AMX + EM	Good
2007^[22]^	46/M	Obesity, smoking, and alcohol abuse	Low back pain and sciatica	L5-S1	*Nocardia asteroides*	Bilateral lumbar incisions fusion	IMP + AMK → RFP	Good
2002^[23]^	54/M	Hypertension, chronic liver disease, alcohol abuse, multiple abscesses	Low back pain, fever, dementia	T11–12, L1–2, and L4	*Nocardia farcinica*	Multiple partial laminectomies and drainage	CIP + SD + FCA	Good
1998^[24]^	39/M	Alcohol abuse	Low back pain, urinary incontinence, fever, vomiting	L4	*Nocardia asteroides*	Laminectomy	TMP/SMZ + AMK + CRO → CRO + SMZ	Good
1994^[25]^	43/M	Laminectomy	Fever, weakness and pain of both low extremities	L3–5	*Nocardia asteroides*	Drainage	AMK + EM → EM	Good
1991^[26]^	65/M	Cirrhosis, COPD, previous MAI pulmonary infection	Leg and low back pain, leg numbness	L4–5	*Nocardia asteroides*	No	SMZ	Good
1991^[27]^	37/M	NA	Fever and low back pain	S	*Nocardia asteroides*	Drainage	TMP-SMZ	Good
1988^[28]^	75/M	Hypertension, history of gardening	Left neck mass, left shoulder and neck pain	C2–4	*Nocardia asteroides*	Drainage	TMP-SMZ → DO	Good
1984^[29]^	53/M	Smoking, alcohol abuse, lumbar surgery, pneumonia	Upper thoracic back pain, weakness of lower extremities	T3–5	*Nocardia asteroides*	Spinal fusion and washout	SD	Good
1984^[30]^	28/M	NA	Cough, fever, diarrhea, lymphadenopathy	C3–5	*Nocardia asteroides*	No	SMZ	Died
1974^[31]^	25/F	Pulmonary tuberculosis	Weakness of lower extremities	T1–2	*Nocardia asteroides*	Laminectomy	NA	Good
1963^[32]^	50/M	History of nocardiosis	Low back pain and upper extremity weakness	C3-T3	*Nocardia asteroides*	Laminectomy,	SMZ + TC	Good
1961^[33]^	38/F	Pneumonia, rural environment, subcutaneous abscess	Fever, cough, paraplegia, bladder disturbances	NA	*Nocardia asteroides*	No	SM + SD	Died

AMK = amikacin, AMX = amoxicillin, CA = cilastatin, CIP = ciprofloxacin, CRO = ceftriaxone, DO = doxycycline, EM = erythromycin, F = female, FCA = fluconazole, IPM = imipenem, M = male, MAI = *Mycobacterium avium*-intracellulare, MH = minocycline, NA = data not available, RFP = rifampin, S = sacrum, SD = sulfadiazine, SM = streptomycin, SMZ = sulfamethoxazole, T = thoracic vertebra, TC = tetracycline, TMP = trimethoprim.

Given that the symptoms, signs, and imaging findings of nocardiosis cannot be generalized, diagnosis of the disease is difficult. To date, 21 reported cases of nocardial spinal osteomyelitis mainly include common types of pathogenic bacteria, but there is a significant difference in antibacterial sensitivity between different types of *Nocardia* spp.^[8]^ Therefore, accurate species-level identification is equally important for the treatment of *Nocardia* spp. With the continuous development of clinical testing methods, a combination of culture and genetic diagnosis has been adopted. The typical growth duration of *Nocardia* spp can be as short as 4 days, but colony development may take several weeks.^[9]^ Therefore, the cultivation time could be appropriately extended to 2 to 4 weeks to improve the detection rate. At present, molecular identification methods have become the gold standard for the detection and identification of *Nocardia* spp.^[10]^ Among them, metagenomic next-generation sequencing is widely used for the identification and detection of *Nocardia* spp because of its advantages in terms of unbiased sampling, strain identification, and drug resistance prediction.^[11]^

Although there remains some controversy regarding resistance to TMP-SMZ,^[10]^ it is still the first-line drug for the treatment of nocardiosis^[12]^ and exhibits good in vitro activity against most species.^[13]^ For cases of TMP-SMZ resistance and severe infections, dual-drug combination therapy based on TMP-SMZ, usually in combination with amikacin, imipenem, or third-generation cephalosporins, is generally recommended.^[14]^ *Nocardia* spp have a slow replication rate and can persist in host cells for a long time. Therefore, the treatment of nocardial osteomyelitis requires long-term antibiotic therapy for 6 months to 1 year.^[14,15]^ After considering the relevant opinions of the laboratory and clinical pharmacy departments, we prescribed a treatment plan for oral TMP-SMZ for 6 months, given the patient’s age and relatively limited site of infection. Unfortunately, the patient completed only 2 months of prescribed treatment. In the subsequent follow-up, the patient’s symptoms gradually improved and after 1 month, the symptoms completely disappeared. At the last follow-up, there was no recurrence and the patient recovered clinically.

This case highlights an individual with isolated lumbar osteomyelitis caused by *N farcinica* infection, which is slightly different from previously reported cases. Insufficient medical testing often makes it difficult to diagnose nocardial infection. Therefore, improving doctors’ understanding of nocardial spinal osteomyelitis and mastering efficient diagnostic and identification methods are of great significance for the early diagnosis and treatment of nocardial spondylitis.

## Acknowledgments

We would like to express our deepest gratitude to our supervisor Professor Wang Zhenyu for his invaluable guidance and support throughout this research.

## Author contributions

**Data curation:** Yuanpeng Yue, Ce Dong, Yihui Liu.

**Formal analysis:** Yuanpeng Yue, Ce Dong, Yihui Liu.

**Investigation:** Yuanpeng Yue, Ce Dong, Yihui Liu.

**Writing** – **original draft:** Yihui Liu.

**Writing** – **review & editing:** Zhenyu Wang.
